# Lumbar Spondylolysis in Juveniles from the Same Family: A Report of Three Cases and a Review of the Literature

**DOI:** 10.1155/2013/272514

**Published:** 2013-09-26

**Authors:** Atsuhisa Yamada, Koichi Sairyo, Isao Shibuya, Ko Kato, Akira Dezawa, Toshinori Sakai

**Affiliations:** ^1^Department of Orthopedic Surgery, Teikyo University Mizonokuchi Hospital, 3-8-3 Mizonokuchi, Takatsu-ku, Kawasaki 213-8507, Japan; ^2^Department of Orthopedic Surgery, University of Tokushima, 3-18-15 Kuramoto-cho, Tokushima, Japan

## Abstract

Spondylolysis is reported as a stress fracture of the pars interarticularis with a strong hereditary basis. Three cases of lumbar spondylolysis in juveniles from the same family are reported, and the genetics of the condition are reviewed. The first boy, a 13-year-old soccer player, was diagnosed with terminal stage L5 bilateral spondylolysis with grade 1 slippage. The second boy, a 10-year-old baseball player, had terminal stage right side unilateral spondylolysis. The third boy, also a 10-year-old baseball player, was diagnosed with early stage bilateral L5 spondylolysis. The second and third boys are identical twins, and all three cases exhibited concomitant spina bifida occulta. Lumbar spondylolysis has a strong hereditary basis and is reported to be an autosomal dominant condition.

## 1. Introduction

In the early 1900s, the pathology of lumbar spondylolysis was considered the congenital failure of fusion of two ossification centers or a hyperflexion injury at birth. In 1957, Wiltse theorized that a bone defect of the pars interarticularis results from the dissolution of continuity of bone due to a congenital weakness [1]. Later in 1975, Witlse et al. stated that lumbar spondylolysis is a fatigue fracture of the pars interarticularis with a strong hereditary basis [2].

Wynne-Davies and Scott analyzed family members including parents, siblings, and children of 35 patients with spondylolysis [3] and found that 14.9% of family members had spondylolysis, which was greater than the prevalence in the general population. They thus concluded that lumbar spondylolysis would have a familial predisposition. After a meticulous review of the pedigrees of the spondylolysis family in 1978, Haukipuro et al. concluded that inheritance of lumbar spondylolysis is autosomal dominant [4]. Then in 1979, Shahriaree et al. came to a similar conclusion [5].

In this paper, we report three cases of lumbar spondylolysis in juveniles under 18 years of age from a single family, and we discuss the genetics of the condition.

## 2. Case Presentation

*Case  1 *(the first boy). A 13-year-old boy was referred to us. Two months earlier, he visited a primary care physician with a complaint of back pain during a sports activity. He was a very active soccer player. Plain radiographs were taken, and L5 spondylolysis was diagnosed. The physician advised him to take a break from sports activity for 2 months until the pain disappeared. The boy was then sent to our sports clinic for specific advice before resuming sports activities.

**Figure 1:**
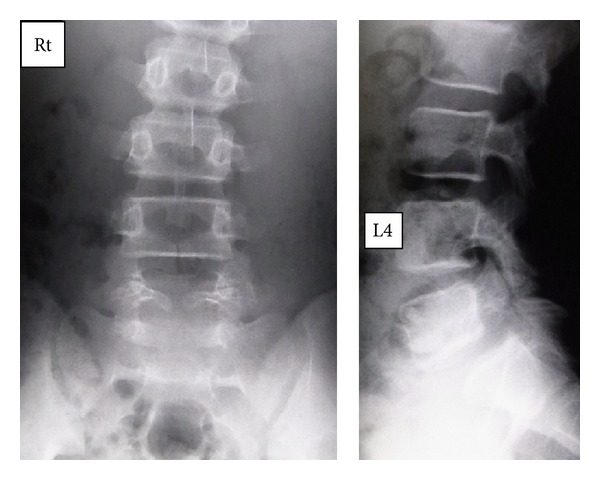
Plain radiographs of Case 1. Spina bifida occulta (SBO) and L5 isthmic olisthesis are obvious.

**Figure 2:**
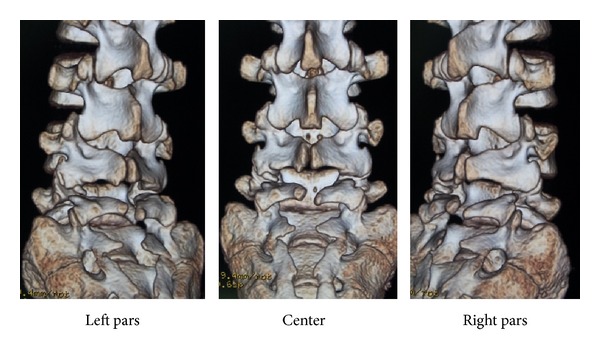
Three-dimensional computed tomography (3D-CT) of Case 1. Note the concomitant existence of L5 bilateral spondylolysis and SBO.

**Figure 3:**
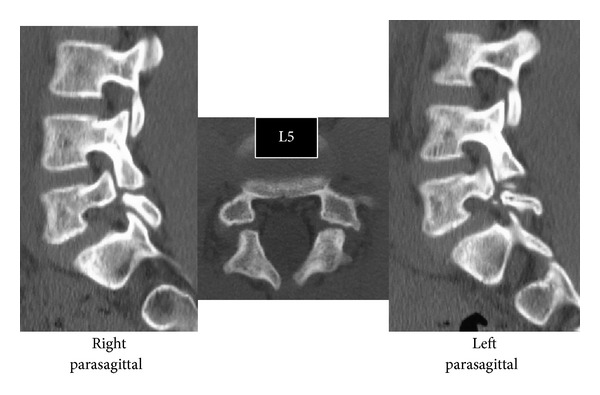
CT of Case 1. L5 spondylolysis is considered to be at the terminal stage (i.e., pseudoarthrosis).

**Figure 4:**
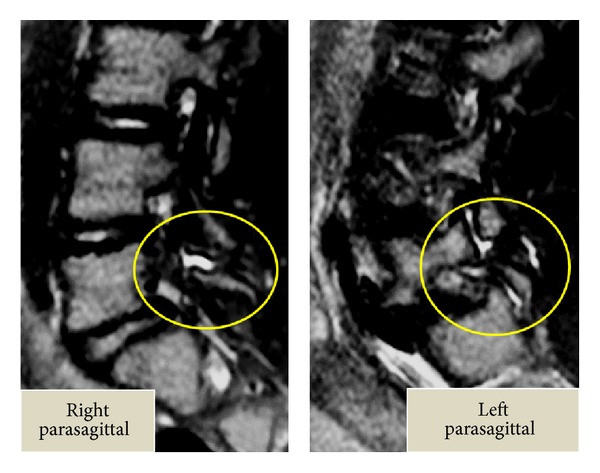
Sagittal short tau inversion recovery magnetic resonance imaging (STIR-MRI) of Case 1. Note the effusion in the pars defects and in the adjacent facet joints. These findings correspond with communicating synovitis.

Slight scoliosis and spina bifida were identified on anteroposterior (Figure 1) radiographs at the first consultation. Grade 1 isthmic spondylolisthesis was noted at L5 with a lumbar index of 80%, and the osseous stage of the vertebra was cartilaginous according to Sairyo's classification [6] since the secondary ossification center of the vertebral body had not ossified yet. Bilateral L5 spondylolysis and spina bifida are clearly observable in Figure 2. Computed tomography (CT) revealed bilateral terminal stage spondylolysis (pseudoarthrosis stage), as proposed by Sairyo et al. [7] (Figure 3). Effusion of the pars defects and adjacent facet joints was evident on sagittal short tau inversion recovery magnetic resonance imaging (STIR-MRI) (Figure 4) findings which correspond to communicating synovitis, the main pathology causing low back pain in terminal stage defects [8].

Since defects at this stage have no chance of bony union with conservative treatment [7, 9], treatment for pain control was selected, and we allowed the patient to resume sports activities. Since the boy's spine was immature and carried a risk of further slippage, follow-up radiographs were planned for 3 times a year until maturation of the vertebral body occurs [6].

*Case  2 *(the second boy). A 10-year-old boy visited a primary care physician with a complaint of back pain during sports activity. He was a very active baseball player. Plain radiography revealed L5 spondylolysis. The physician advised him to take a break from sports activity for 2 months until the pain disappeared. He was sent to our sports clinic for specific advice before resuming sports activities.

**Figure 5:**
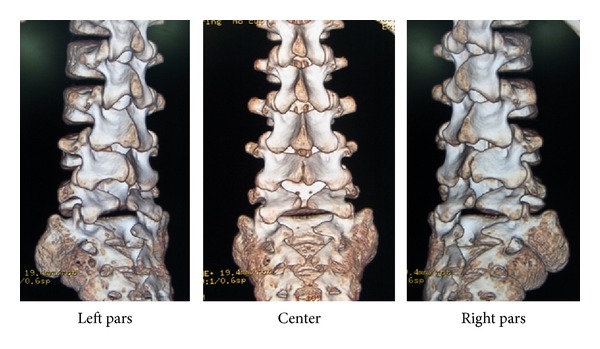
3D-CT of Case 2. Note the concomitant existence of the right side L5 unilateral spondylolysis and SBO.

**Figure 6:**
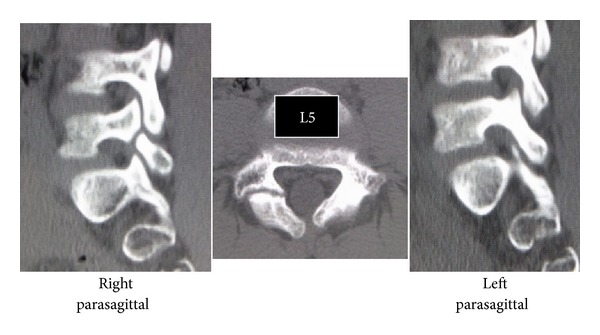
CT of Case 2. The right side unilateral L5 spondylolysis is considered to be at the terminal stage.

Terminal stage unilateral L5 spondylolysis (pseudoarthrosis) of the right side and spina bifida were clearly observed on CT (Figures 5 and 6). Since defects at this stage have no chance of bony union with conservative treatment [7, 9], treatment for pain control was selected, and we allowed the patient to resume sports activities. Unilateral spondylolysis has a low chance of further slippage [10]; therefore, we recommended the boy to visit us if back pain recurred.

*Case  3 *(the third boy). A 10-year-old male, who was an identical sibling of the second boy, visited us with a one-week history of back pain during baseball activity. He was also a very active baseball player. Since both his siblings had lumbar spondylolysis, his parents took him directly to our sports clinic with a concern of the same condition.

**Figure 7:**
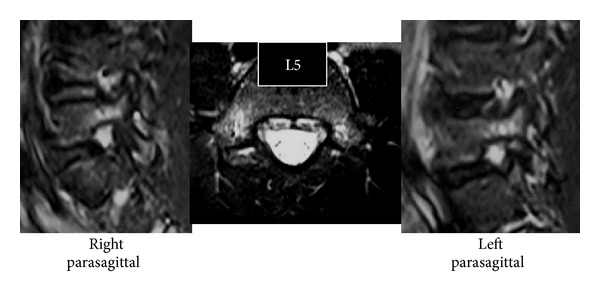
STIR-MRI of Case 3. Note the high signal changes of the pedicle, indicating marrow edema. This is a sign that the adjacent pars would have an early stage stress fracture of the pars interarticularis.

**Figure 8:**
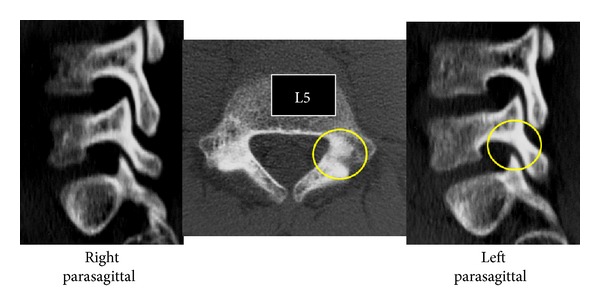
CT of Case 3. The left pars shows bony absorption, indicating the beginning of a stress fracture of the pars.

**Figure 9:**
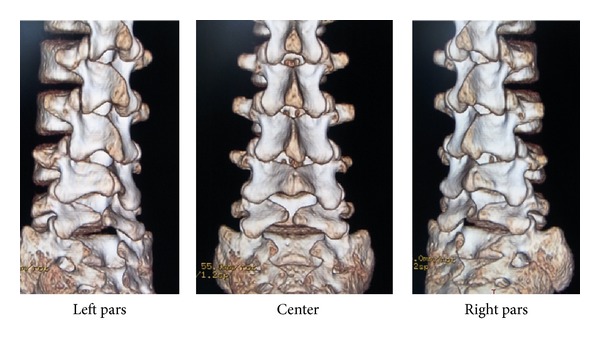
3D-CT of Case 3. Note the concomitant SBO.

STIR-MRI of the bilateral pedicle showed high signal changes which indicated an adjacent pars stress fracture at the early stage (Figure 7), according to the staging of Sairyo et al. [11]. CT indicated slight bone absorption at the ventral aspect of the left pars (Figure 8). Because the stress reaction of early spondylolysis initially appears at this site [12], MRI and CT findings strongly suggested the initial stage of spondylolysis. To avoid complete fracture, we applied a molded thoracolumbosacral brace and restricted participation in sports for 3 months. For spondylolysis at this stage, conservative treatment has been reported to be effective [9]. Like both his other siblings, spina bifida was revealed on three-dimensional CT (Figure 9).

## 3. Discussion

**Figure 10:**
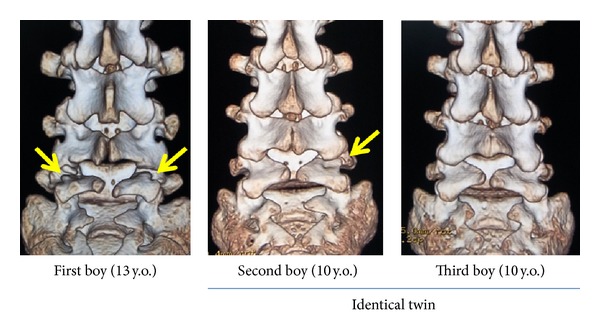
3D-CT of Cases  1–3, all showing similar structures.

In the past, the pathology of lumbar spondylolysis was considered the congenital failure of fusion of two ossification centers or hyperflexion injury at birth. However, following Wiltse's proposal in 1957 [1], the pathomechanism of spondylolysis shifted to a consensus of fatigue fracture of the pars interarticularis with a strong hereditary basis. Here, we presented three cases of different types of lumbar spondylolysis at different stages in three juvenile brothers. The three-dimensional CT images of the cases are shown in Figure 10. The first boy had bilateral L5 spondylolysis at the terminal stage with grade I slippage. The second boy had a unilateral terminal defect at L5 of the right side. The third boy had a very early stage bilateral spondylolysis (i.e., the beginning of a stress fracture). The presence of lumbar spondylolysis in all siblings in the present study supports the concept of the “spondylolysis gene” proposed by Haukipuro et al. in 1978 [4].

The genetic correlation between lumbar spondylolysis and spina bifida occulta (SBO) has been discussed for a long time. In 1962, Wiltse stated that SBO is 13-fold more frequent in patients with spondylolysis compared with the general population [13]. The prevalence of SBO was reported by Taillard as 42% [14], by Laurent and Einole as 22% [15], and by Meyerding as 35% [16]. In a review of 500 children at the age of 6 years, Fredrickson et al. in 1984 found a lumbar spondylolysis prevalence of 4.4% and a SBO prevalence of 59% [17]. All three cases showed SBO from L5 to the sacrum, as shown in Figure 10. To date, however, there have been no studies on the genetic contribution of SBO to spondylolysis. Ucar et al. [18] reported a significant coexistence of SBO and dysplasia of the hip joint. Although they did not conduct a genetic study, it can be assumed that patients with SBO have a genetic weakness in ossification or bone metabolism. Likewise, patients with SBO are likely to have stress fractures. Recently, genetic approaches to clarify an unfused neural canal have been conducted [19, 20]. Further detailed genetic research on SBO will help to clarify the genetic predisposition of coexisting disorders such as spondylolysis and may shed further light on the “spondylolysis gene.”

Roche and Rowe [21] reported a different incidence of lumbar spondylolysis between races and sex (white males: 6.4%; African American males: 2.8%; white females: 2.3%; African American females: 1.1%). Sakai et al. [10] reviewed 2000 CT scans taken for abdominal disease, not for back problems, and found the prevalence of spondylolysis to be 7.9% for males and 3.9% for females in the Japanese general population. Such differences between races and sex are in good agreement with the notion that lumbar spondylolysis has an underlying genetic influence.

The highest prevalence of spondylolysis (54%) was reported in an Inuit tribe where Simper [22] found the condition in 24 out of 46 spines from native residents of Greenland. Similarly, other reports confirm the high prevalence of spondylolysis in other Inuit tribes [23–25]. It is not difficult to assume that a genetic study in an area with a high prevalence of lumbar spondylolysis may hold the key to uncovering the “spondylolysis gene.”
